# Efficient Identification of *Pulsatilla* (Ranunculaceae) Using DNA Barcodes and Micro-Morphological Characters

**DOI:** 10.3389/fpls.2019.01196

**Published:** 2019-10-09

**Authors:** Qiu-jie Li, Xi Wang, Jun-ru Wang, Na Su, Ling Zhang, Yue-ping Ma, Zhao-yang Chang, Liang Zhao, Daniel Potter

**Affiliations:** ^1^College of Life Sciences, Northwest A&F University, Yangling, China; ^2^Herbarium of Northwest A&F University, Yangling, China; ^3^College of Life Sciences, Tarim University, Alaer, China; ^4^College of Life and Health Sciences, Northeastern University, Shenyang, China; ^5^Department of Plant Sciences, MS2, University of California, Davis, Davis, CA, United States

**Keywords:** barcoding markers, ITS, *pulsatilla*, ranunculaceae, species identification

## Abstract

*Pulsatilla* (Ranunculaceae) comprises about 40 species, many of which have horticultural and/or medicinal importance. However, the recognition and identification of wild *Pulsatilla* species is difficult due to the presence of complex morphological characters. DNA barcoding is a powerful molecular tool capable of rapidly and accurately distinguishing between species. Here, we assessed the effectiveness of four commonly used DNA barcoding loci—*rbcL* (R), *trnH-psbA* ( T ), *matK* (M), and ITS (I)—to identify species of *Pulsatilla* from a comprehensive sampling group. Among the four barcoding single loci, the nuclear ITS marker showed the highest interspecific distances and the highest rate of correct identification. Among the eleven combinations, the chloroplast multi-locus R+T and R+M+T combinations were found to have the best species discrimination rate, followed by R+M. Overall, we propose that the R+M+T combination and the ITS marker on its own are, respectively, the best multi- and single-locus barcodes for discriminating among species of *Pulsatilla*. The phylogenetic analysis was able to distinguish species of *Pulsatilla* to the subgenus level, but the analysis also showed relatively low species resolution. This may be caused by incomplete lineage sorting and/or hybridization events in the evolutionary history of the genus, or by the resolution limit of the candidate barcodes. We also investigated the leaf epidermis of eight representative species using scanning electronic microscopy. The resulting micro-morphological characters were valuable for identification of related species. Using additional genome fragments, or even whole chloroplast genomes combined with micro-morphological data may permit even higher resolution of species in *Pulsatilla*.

## Introduction

**Figure 1:**
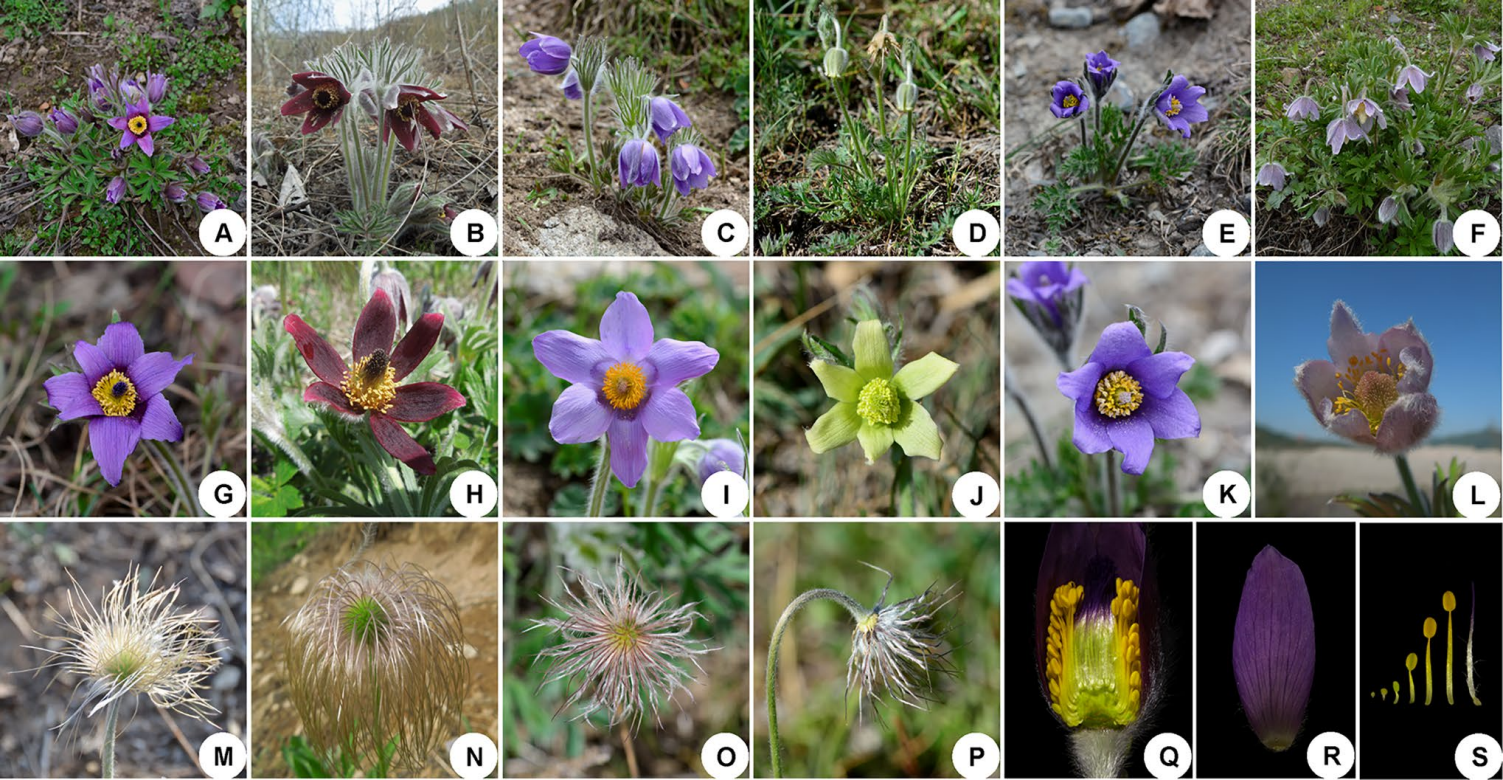
Representatives of species illustrating the morphological variation and similarities in *Pulsatilla*. **(A–F)** plants in flower; **(G–L)** anthetic flower. **(A)** *P. chinensis*; **(B)** *P. cernua*; **(C)** *P. patens*; **(D)** *P. camoanella*; **(E)** *P. ambigua*; **(F)** *P. dahurica*; **(G)** *P. chinensis*; **(H)** *P.cernua*; **(I)** *P. patens*; **(J)** *P. camoanella*; **(K)** *P. ambigua*; **(L)** *P. dahurica*; **(M–P)** style strongly elongate and plumose in fruit; **(Q)** lateral view of flower showing the retarded stamen in outermost; **(R)** sepal; **(S)** stamens and pistil.

The Ranunculaceae is a large and complex plant family, including approximately 59 genera and 2,500 species (Tamura, 1995). *Pulsatilla* Miller, first described in 1753, consists of about 40 species that are restricted to temperate subarctic and mountainous areas in the Northern Hemisphere (Tamura, 1995). Plants of *Pulsatilla* species are often covered with long, soft hairs. Their flowers are solitary and bisexual, with three bracts forming a bell-shaped involucre. The tepal number is always six, and stamens are generally numerous, with the outermost ones resembling degenerated petals (although *Pulsatilla kostyczewii* is a notable exception to this tendency) (**Figure 1**; Wang et al., 2001; Ren et al., 2011; Ren et al., 2015).

Most authors have treated *Pulsatilla* as a subgenus or section of the genus *Anemone* s.l. (Linnaeus, 1753; Endlicher, 1839; Tamura, 1967; Tamura, 1995; Hoot et al., 1994). However, Miller (1754), Adanson (1763), and Wang et al. (2001) have supported a model that separates *Pulsatilla* from *Anemone* as an independent genus. Recent phylogenetic studies have shown that all species within *Pulsatilla* are clustered in a monophyletic group, which is nested within *Anemone* (Hoot et al., 1994; Jiang et al., 2017). Morphologically, *Pulsatilla* can easily be distinguished from *Anemone* s.s., since species of the former have a long, plumose beak on the achenes formed by the persistent style and stamens (Tamura, 1995; Wang et al., 2001) whereas species of the latter do not. Because the primary goal of the present study is to test the use of DNA barcodes for species in the *Pulsatilla* clade, we here follow the treatment of Wang et al. (2001) and Grey-Wilson (2014), regarding *Pulsatilla* as a distinct genus.

There are eleven species of *Pulsatilla* found in China, most of which are found primarily in the northern part of the country (Grey-Wilson, 2014). Some species of *Pulsatilla* have been used in traditional Chinese medicine for many years for “blood-cooling” or “detoxification” (Pharmacopoeia, 2015). In particular, the root of *Pulsatilla chinensis* (Bunge) Regel is a well-known ingredient included in the Chinese Pharmacopoeia (2015). Many species (e.g. *P. ambigua*, *P. campanella*, *P. cernua*, *P. chinensis*, *P. dahurica* and *P. turczaninovii*) used in folk medicine have been found to contain pharmacologically useful chemical components, including those with anti-cancer and anti-inﬂammatory activities (Xu et al., 2012; Wang et al., 2016; Suh and An 2017). The contents of these components differ in various species, resulting in different clinical pharmacological effects. Thus, in cases where target species can be easily confused with their close relatives, undesired species can be inadvertently collected, resulting in negative effects on drug efficacy and patient safety, as has been shown in other plant groups of medicinal importance in China (Zhang et al., 2015a; Zhang et al., 2015b).

*Pulsatilla* is an especially challenging, complex group. In all treatments published to date, the genus has been treated as comprising two to four subgenera: subgenus *Miyakea*, which contains only one species, *P. integrifolia*; subgenus *Kostyczewianae*, which has only one species, located in Central Asia and northwestern China; subgenus *Preonanthus*, which includes six species; and the largest subgenus *Pulsatilla*, which comprises 29 species (Tamura, 1995; Wang et al., 2001; Grey-Wilson, 2014; Sramkó et al., 2019). However, *Pulsatilla* shows a frustratingly complicated pattern of intrageneric morphological variability (Grey-Wilson, 2014). The recognition and identification of wild *Pulsatilla* species based on traditional approaches is difficult due to transitional intraspecific morphological characteristics in many *Pulsatilla* species. For instance, *P. turczaninovii* and *P. tenuiloba* were considered to be two separate species that could be told apart by the number of pairs of lateral leaflets (i.e. a leaf blade with 3 or 4 pairs of lateral leaflets vs. a leaf blade with 5 or 6 pairs of lateral leaflets) (Wang et al., 2001). After carefully checking specimens and population investigation, we found that the leaflet numbers of *P. turczaninovii* and *P. tenuiloba* are overlapping, and some individuals have both 4 and 5 (even 6) pairs of lateral leaflets. Flowers nodding before anthesis is recorded as a diagnostic character of *P. campanella*, but this character was also found in *P. ambigua*, *P. cernua* and *P. dahurica*, and their flower colors show continuous transitional shades of blue (Wang et al., 2001). Thus, these characters are not reliable and make *Pulsatilla* difficult to identify.

DNA barcoding aims to achieve rapid and accurate species recognition by sequencing short DNA sequences or a few small DNA regions (Fay et al., 1997; Hebert et al., 2003a; Hebert and Gregory, 2005; Kress et al., 2005; China Plant BOL Group, 2011; Kress, 2017). This technology was first developed to identify animal species; for example, Hebert et al. (2003b) argued that “the mitochondrial gene cytochrome *c* oxidase I (*COI*), can serve as the core of a global bio-identification system for animals”. Studies have continued to demonstrate that the *COI* gene fragment efficiently discriminates among animal species, including amphibians (Vences et al., 2005), birds (Hebert et al., 2004; Yoo et al., 2006; Kerr et al., 2007; Tavares and Baker, 2008), fish (Yancy et al., 2008; Ward et al., 2009), and insects (Hajibabaei et al., 2006; Pons et al., 2006). In plants, however, frequent recombination and low mutation rates restrict the utility of mitochondrial barcode markers (Cho et al., 1998; Cho et al., 2004). The search for suitable candidates has therefore focused on chloroplast and nuclear DNA markers (De Salle et al., 2005; Kress et al., 2005; CBOL Plant Working Group, 2009; China Plant BOL Group, 2011; Yan et al., 2015), although such markers are not always easy to amplify and sequence in all plant taxa using universal primers. Numerous studies have suggested that four standard barcodes — three from the chloroplast genome [the ribulose-bisphosphate/carboxylase Large-subunit gene (*rbcL*), the maturase-K gene (*matK*), and the *trnH-psbA* intergenic spacer and the nuclear ribosomal internal transcribed spacers (nrITS)] — should be used as core barcode markers for the molecular identification of plants (China Plant BOL Group, 2011; Xiang et al., 2011; Zhang et al., 2012; Li et al., 2015).

Significant progress has been made in DNA barcoding in plants (Zhang et al., 2015b; Han et al., 2016; Li et al., 2016). However, the discrimination of closely related species using only molecular data is still a major challenge in some genera (Piredda et al., 2011; Zhang et al., 2012; Chen et al., 2015; Guo et al., 2015). Morphological characters, including the shape of nutritive and reproductive organs, remain highly valuable for plant identification and studies of plant evolution (Ronse De Craene, 2010). Micromorphological characters have been shown to have great value for species identification and systematics (i.e., Kong, 2001; Ren et al., 2003), and these have rarely been considered by previous barcode studies. However, the combination of morphological data and DNA barcodes may be essential for species discrimination, especially in closely related species (Zhang et al., 2012; Li et al., 2016).

Previous molecular phylogenetic studies have included few species from the genus *Pulsatilla* (Hoot et al., 1994; Hoot, 1995; Ehrendorfer and Samuel, 2001; Schuettpelz et al., 2002; Ehrendorfer et al., 2009; Meyer et al., 2010; Kylem et al., 2010; Hoot et al., 2012; Mlinarec et al., 2012a; Mlinarec et al., 2012b; Jiang et al., 2017). In a recent phylogenetic study of *Pulsatilla*, few species were from Asia and few individuals were collected for one species (Sramkó et al., 2019). Obtaining DNA barcode data from a dataset created by comprehensive sampling of a taxonomically difficult genus such as *Pulsatilla* should contribute to understanding the discriminatory potential of barcodes in morphologically complex clades. The establishment of an available barcoding system for *Pulsatilla* may also facilitate further utilization of these taxa, as well as further research into their taxonomy.

In this study, four DNA barcode regions (*rbcL*, *matK*, *trnH-psbA*, and ITS) were assessed in 19 species (representing three subgenera) of *Pulsatilla*. Approximately 50% of the accepted species of *Pulsatilla* found in Europe and the Americas were included, as were 90% of the species found in China (Tamura, 1995; Grey-Wilson, 2014; Wang et al., 2001). Our objectives were to: test the effectiveness of common core DNA barcodes (*rbcL*+*matK*) in *Pulsatilla*, evaluate the resolution of these four barcodes, and use 2- to 4-region combinations to correctly identify individuals. We also aimed to develop a protocol that could effectively discriminate among closely related species, primarily for species discrimination of medicinal plants. In addition, we added micro-morphological analyses of leaf tissue obtained using scanning electronic microscopy (SEM) to reveal the taxonomic relationships among *Pulsatilla*.

## Materials and Methods

In total, 52 accessions representing 19 *Pulsatilla* species (including widely used medicinal species) were involved in this study (**Table 1**). This sample covered each of the three subgenera from Asia, Europe, and America. Nine samples were sourced from herbarium specimens, while 43 samples were newly collected. All samples were taxonomically identified using published floras, monographs, and references. In total, one to five individuals per species were sampled from different populations in the wild. Fresh leaves were dried in silica gel upon collection and the longitude, latitude, and altitude of each collection site (population) were recorded using a GPS unit (**Table 1**). Voucher specimens were stored in the Herbarium of Northwest A&F University (WUK) and the US National Herbarium (US). Singleton species (species represented by one individual) were only used as potential causes of failed discrimination, and were not included in the calculation of the identification success rate. Three members of *Anemone*, two of *Clematis*, one of *Anemoclema*, and one of *Hepatica* were selected as outgroups for tree-based analyses.

**Table 1:** Voucher information and GenBank accession numbers for *Pulsatilla* and outgroups sampled in this study. Classification follows Grey-Wilson (2014) and Tamura (1995). All voucher specimens are deposited in the Northwest A&F University Herbarium (WUK) and the US National Herbarium (US).

Taxon	Number	Voucher	Location	matK	rbcL	trnH-psbA	ITS	Latitude (N)	Longitude (E)	Alt. (m)
*Pulsatilla alba*	101	s.n.	Italy. Valle d’Aosta	MK341992	MK341971	MK341913	MK341853	–	–	–
*Pulsatilla alpina*	104	s.n.	Graubunden	MK341987	MK341970	MK341914	MK341852	–	–	–
*Pulsatilla ambigua*	107	ZL-20140519-01	China. Xinjiang Urumchi	MK342022	MK341964	MK341895	MK341821	N43°13.509′	E087°07.906′	2,122m
*Pulsatilla ambigua*	108	ZL-20140519-02	China. Xinjiang Urumchi	MK342021	MK341963	MK341894	MK341803	N43°13.509′	E087°07.906′	2,122m
*Pulsatilla ambigua*	109	ZL-20140519-03	China. Xinjiang Urumchi	MK342020	MK341962	MK341893	MK341820	N43°13.509′	E087°07.906′	2,122m
*Pulsatilla ambigua*	175	ZL-20140519-04	China. Xinjiang Urumchi	MK342019	MK341961	MK341892	MK341823	N43°13.509′	E087°07.906′	2,122m
*Pulsatilla ambigua*	189	ZL-20140519-05	China. Xinjiang Urumchi	MK342018	MK341960	MK341891	MK341822	N43°13.509′	E087°07.906′	2,122m
*Pulsatilla camanella*	111	ZL-20140525-01	China. Xinjiang Zhaosu	MK341985	MK341968	MK341873	MK341819	N43°29.382′	E081°06.913′	1,827m
*Pulsatilla camanella*	112	ZL-20140525-02	China. Xinjiang Zhaosu	MK341984	MK341967	MK341872	MK341818	N43°29.382′	E081°06.913′	1,827m
*Pulsatilla camanella*	113	ZL-20140525-03	China. Xinjiang Qapqal Xibe	MK341983	MK341966	MK341871	MK341817	–	–	–
*Pulsatilla camanella*	114	ZL-20140525-04	China. Xinjiang Qapqal Xibe	MK341982	MK341965	MK341870	MK341816	–	–	–
*Pulsatilla cernua*	115	ZL-20140421	China. Liaoning Huanren	MK342016	MK341929	MK341898	MK341836	N41°18.075′	E124°53.928′	800m
*Pulsatilla cernua*	116	ZL-20140531-01	China. Jilin Dongchang	MK342015	MK341928	MK341897	MK341834	–	–	–
*Pulsatilla cernua*	176	ZL-20140531-02	China. Jilin Tonghua	MK342014	MK341927	MK341877	MK341833	–	–	–
*Pulsatilla cernua*	177	ZL-20140511	China. Jilin Tonghua	MK342013	MK341926	MK341876	MK341835	–	–	–
*Pulsatilla cernua*	190	ZL-20090501	China. Jilin Tonghua	MK342012	MK341925	MK341896	MK341832	–	–	–
*Pulsatilla chinensis*	119	ZL-20140406	China. Shaanxi Taibai	MK342028	MK341934	MK341869	MK341824	N34°18.072′	E107°11.880′	–
*Pulsatilla chinensis*	121	ZL-20140502	China. Jilin Erdao	MK342027	MK341933	MK341890	MK341827	–	–	–
*Pulsatilla chinensis*	122	ZL-20140701	China. Liaoning Huludao	MK342026	MK341932	MK341875	MK341826	N47°47.811′	E120°51.223′	82m
*Pulsatilla chinensis*	178	ZL-20140729	China. Liaoning Chaoyang	MK342024	MK341930	MK341874	MK341825	–	–	–
*Pulsatilla dahurica*	128	ZL-20140602	China. Jilin Yitong	MK342025	MK341931	MK341889	MK34183	N43°34.219′	E125°13.337′	412m
*Pulsatilla dahurica*	129	ZL-20140718	China. Inner Mongolia Argun	MK342011	MK341959	MK341867	MK341842	–	–	–
*Pulsatilla dahurica*	130	ZL-20140517	China. Jilin Liuhe	MK342010	MK341958	MK341866	MK341841	N42°04.550′	E126°05.167′	568m
*Pulsatilla dahurica*	131	ZL-20140602	China. Jilin Panshi	MK342009	MK341957	MK341865	MK341840	N43°53.055′	E125°44.443′	232m
*Pulsatilla dahurica*	174	YJL_s.n.	China. Jilin	MK342008	MK341951	MK341864	MK341839	–	–	–
*Pulsatilla grandis*	132	s.n.	Moai Juni	MK342023	MK341956	MK341912	MK341849	–	–	–
*Pulsatilla hirsutissima*	133	Ramaly Spotls 15944	US	MK341999	MK341940	MK341900	MK341848	–	–	–
*Pulsatilla kostyczewii*	135	s.n.	China. Xinjiang Ucha	MK341979	MK341922	MK341863	MK341802	–	–	–
*Pulsatilla latifolia*	136	0044781	USSR	MK341998	MK341941	MK341911	MK341828	–	–	–
*Pulsatilla ludoviciana*	137	Aven_Nelson_4305	US. Albany	MK341997	MK341939	MK341899	MK341847	–	–	–
*Pulsatilla occidentalis*	145	Pound C Alatchison 943	US. Calfornia	MK341986	MK341969	MK341868	MK341851	–	–	–
*Pulsatilla patenssubsp. multifida*	146	ZL-20140717a01	China. Inner Mongolia Argun	MK341995	MK341938	MK341910	MK341846	N51°31.939′	E120°02.615′	509.4m
*Pulsatilla patenssubsp. multifida*	147	ZL-20140717a02	China. Inner Mongolia Argun	MK341994	MK341937	MK341909	MK341845	N51°31.939′	E120°02.615′	509.4m
*Pulsatilla patenssubsp. multifida*	179	ZL-20140717a03	China. Inner Mongolia Argun	MK341993	MK341936	MK341908	MK341844	N51°31.939′	E120°02.615′	509.4m
*Pulsatilla patenssubsp. multifida*	180	ZL-20140717a04	China. Inner Mongolia Argun	MK341988	MK341935	MK341907	MK341843	N51°31.939′	E120°02.615′	509.4m
*Pulsatilla patens*	148	ZL-20140521	China. Xinjiang Fuyun	MK341991	MK341955	MK341903	MK341838	N47°43.418′	E089°19.436′	1,492m
*Pulsatilla patens*	149	ZL-20140522	China. Xinjiang Altay	MK341996	MK341954	MK341902	MK341829	N48°00.298′	E088°19.069′	1,993m
*Pulsatilla patens*	150	ZL-20140523-01	China. Xinjiang Burqin	MK341990	MK341953	MK341901	MK341837	N47°42.360′	E086°51.480′	
*Pulsatilla patens*	151	ZL-20140523-02	China. Xinjiang Habahe	MK341989	MK341952	MK341878	MK341830	N48°29.816′	E087°08.545′	1,464m
*Anemone reflexa*	167	ZL-20140406	China. Shaanxi Taibai	MK341978	MK341921	MK341861	MK341854	–	–	–
*Pulsatilla sukaczevii*	181	ZL-20180512-01	China. Inner Mongolia Hohhot	MK341981	MK341924	MK341888	MK341815	N41°12.833′	E111°39.917′	1,653m
*Pulsatilla sukaczevii*	182	ZL-20180512-02	China. Inner Mongolia Hohhot	–	–	MK341887	MK341814	N41°12.833′	E111°39.917′	1,653m
*Pulsatilla sukaczevii*	187	ZL-20180512-03	China. Inner Mongolia Hohhot	–	–	MK341886	MK341813	N41°12.833′	E111°39.917′	1,653m
*Pulsatilla sukaczevii*	188	ZL-20180512-04	China. Inner Mongolia Hohhot	MK341980	MK341923	MK341885	MK341812	N41°12.833′	E111°39.917′	1,653m
*Pulsatilla tenuiloba*	159	ZL-20140717b01	China. Inner Mongolia Argun	MK342007	MK341950	MK341906	MK341808	N51°16.517′	E119°59.456′	552.1m
*Pulsatilla tenuiloba*	160	ZL-20140717b02	China. Inner Mongolia Argun	MK342006	MK341949	MK341884	MK341810	N51°16.517′	E119°59.456′	552.1m
*Pulsatilla tenuiloba*	183	ZL-20140717b03	China. Inner Mongolia Argun	MK342002	MK341948	MK341883	MK341807	N51°16.517′	E119°59.456′	552.1m
*Pulsatilla tenuiloba*	184	ZL-20140717b04	China. Inner Mongolia Argun	MK342005	MK341947	MK341882	MK341806	N51°16.517′	E119°59.456′	552.1m
*Pulsatilla turczaninovii*	161	ZL-20140718-01	China. Inner Mongolia Argun	MK342004	MK341946	MK341881	MK341811	N51°16.517′	E119°59.456′	552.1m
*Pulsatilla turczaninovii*	162	ZL-20140718-02	China. Inner Mongolia Argun	MK342003	MK341945	MK341905	MK341809	N51°16.517′	E119°59.456′	552.1m
*Pulsatilla turczaninovii*	185	ZL-20140718-03	China. Inner Mongolia Argun	MK342001	MK341944	MK341880	MK341805	N51°16.517′	E119°59.456′	552.1m
*Pulsatilla turczaninovii*	186	ZL-20140718-04	China. Inner Mongolia Argun	MK342000	MK341943	MK341879	MK341804	N51°16.517′	E119°59.456′	552.1m
*Pulsatilla vulgaris*	166	ZL-20170919	China. Shaanxi Xi’an (cult.)	MK342017	MK341942	MK341904	MK341850	N34°12.867′	E108°57.915′	–
*Anemone vitifolia*	169	ZL-20110715	China. Shaanxi Taibai	MK341973	MK341917	MK341862	MK341855	–	–	–
*Anemone demissa*	168	ZL-20140810	China. Yunnan Shangri-la	MK341972	MK341915	MK341856	–	–	–	–
*Clematis hexapetala*	170	ZL-20140711	China. Inner Mongolia Argun	MK341976	MK341918	MK341860	MK341800	–	–	–
*Clematis tangutica*	171	ZL-20140829	China. Qinghai Tongren	MK341975	MK341919	MK341859	MK341799	–	–	–
*Hepatica nobilis*	172	ZL-20150501	US. Washington	MK341977	MK341916	MK341857	–	–	–	–
*Anemoclema glaucifolium*	173	LHN-2011101	China. Yunnan Shangri-la	MK341974	MK341920	MK341858	MK341798	–	–	–

### DNA Extraction, PCR Amplification, and Sequencing

Total genomic DNA from freshly collected samples was extracted from approximately 20 mg of silica-dried leaves using a modified cetyltrimethylammonium bromide (CTAB) protocol (Ma and Dai, 2009). For herbarium specimens, we extracted DNA using DNeasy Plant mini kits (QIAGEN, Guangzhou, China). Amplification of DNA regions was performed by standard polymerase chain reaction (PCR). The primer sequences and thermocycling conditions for PCR amplification, are listed in **Table 2**. PCR reactions were conducted in 25 µl reaction volumes containing 12.5 µl 2 × Taq PCR mix (CWBIO, Xi’an, China), 1.0 µl of each primer (10 µmol/µl), 10.5 µl ddH_2_O, and 1.0 µl template DNA (30–50 ng). PCR products were run on 1% agarose gels to check whether PCR products showed a clear single band. For those PCR products that did not show a clear single band, the corresponding template DNA was amplified again with two/one pair segmented primers (**Table 2**, **S1**, **S2**). High-quality PCR products wereSequencing was performed from both directions to reduce sequencing error.

**Table 2:** List of primers for candidate barcodes.

Region	Primer	Sequence (5′–3′)	References
rbcL	1F	ATGTCACCACAAACAGAAAC	Fay et al. (1997)
	R	TCACAAGCAGCTAGTTCAGGACTC	Asmussen and Chase (2001)
matK	390F	CGATCTATTCATTCAATATTTC	Cuénoud et al. (2002)
	1326R	TCTAGCACACGAAAGTCGAAGT	Cuénoud et al. (2002)
ITS	5a F	CCTTATCATTTAGAGGAAGGAG	Stanford et al. (2000)
	4R	TCCTCCGCTTATTGATATGC	Stanford et al. (2000)
trnH-psbA	trnH2R	CGCGCATGGTGGATTCACAATCC	Tate and Simpson (2003)
	psbAF	GTTATGCATGAACGTAATGCTC	Sang et al. (1997)

### Data Analysis

All sequence assemblies and adjustments were performed using Geneious v.9.0 (Kearse et al., 2012). Sequences were aligned with MUSCLE (Edgar, 2004). In particular, the number of indel and variable sites events for each dataset was inferred by deletion/insertion polymorphism (DIP) and polymorphic site analyses performed by DnaSP v5 (Librado and Rozas, 2009). To assess the barcoding resolution for all barcodes (*rbcL*, *matK*, *trnH-psbA*, ITS, and combinations of these), three analytical methods were employed. These included the pair-wise genetic distance method (PWG-distance), the sequence similarity method (TAXONDNA), and phylogenetic-based methods (NJ, BI, and ML). Each of these analyses is described in detail below.

### PWG-Distance Method

For the pair-wise genetic distance-based method, five parameters — i.e. average distance, average interspecific distance, average intraspecific distance, smallest interspecific distance, and largest intraspecific distance — were calculated in MEGA 7 using the Kimura two-parameter distance model (K2P), to explore intra- and inter-species variation (Kumar et al., 2016).

Furthermore, to assess the candidate barcodes for the PWG-distance method, two analytical computations were made; i.e. the barcoding gap between interspecific and intraspecific distances and the local barcoding gap for species resolution. The barcoding gap was used to test for appropriate barcode markers, which show high interspecific but low intraspecific genetic divergence (Han et al., 2016). We graphed the distribution of intra- and inter-specific divergence of each candidate barcode with their combinations to show the barcoding gap. Next, we graphed the local barcoding gap to reveal the species resolution power of candidate barcodes. We considered discrimination to be successful if the smallest interspecific distance, involving more than one individual for one species, was larger than its largest intraspecific distance.

### Sequence Similarity Method

We used the proportion of correct identifications to assess the potential of all markers for accurate species identification with TAXONDNA (Species Identifier 1.8 program). The “Best Match” (which assigns queries to species with the best-matching sequences, regardless of their similarity), and “Best Close Match” (which assigns queries to species if a threshold similarity is met) tests in TAXONDNA were run for all species that were represented by more than one individual (Meier et al., 2006).

### Phylogeny-Based Methods

To evaluate the species discrimination power of the four single barcoding markers, three different tree-building analyses—i.e. the Neighbor Joining (NJ) tree, Bayesian inference (BI) tree, and Maximum likelihood (ML) analyses—were used. The NJ and ML analyses of all markers were conducted by K2P model using MEGA7. For the BI analysis, best substitution models were selected according to the Akaike information criterion (AIC) by jModeltest version 2.1.7 (Posada and Buckley 2004; Posada, 2008; Darriba et al., 2012). BI trees were conducted in MrBayes v 3.1 (Huelsenbeck and Ronquist, 2001; Ronquist and Huelsenbeck, 2003; Kumar et al., 2016). The Markov chain Monte Carlo (MCMC) analysis was run for 10,000,000 generations. The first 25% of the generations were discarded as burn-in after checking for stationarity and convergence of the chains, and a consensus tree was constructed using the remaining trees. Generally, species forming separate clusters in the tree with bootstrap support >50% were considered to be distinct.

### Scanning Electron Microscopy Observation of Leaves

Leaf collection information for *Pulsatilla* species is shown in **Table 1**. Leaves from 8 species, which were difficult to identify by barcode, were fixed in FAA (Formalin: acetic acid: ethanol: water = 10:5:50:35). The materials were first dissected and dehydrated in an ethanol and iso-amyl acetate series. Next, they were subjected to critical-point drying in CO_2_, sputter-coating with gold, and imaged using a HITACHI S-3500 scanning electron microscope (SEM). The backgrounds of SEM images were edited and details were colored using Adobe Photoshop. Photographs of mature leaves were taken with a Nikon D7100 digital camera against a black background. Descriptions of leaf morphology were based on 30 mature leaves.

## Results

### Amplification and Sequence Analysis

The characteristics of the four DNA barcoding regions are shown in **Table 3**. For each of the four DNA barcoding regions (*rbcL*, *matK*, *trnH-psbA*, and ITS), PCR amplification and sequencing using a universal primer pair had a high success rate — i.e. 96.15, 96.15, 100, and 100%, for *rbcL*, *matK*, *trnH-psbA*, and ITS, respectively. It was difficult to obtain target barcodes for herbarium samples and the success rates from these samples were low. We could not get target barcodes for the 80% of the herbarium samples even when segmented primer pairs were used. A total of 232 new sequences were obtained from 52 accessions, which included 57 sequences for each of *rbcL* and *matK*, and 59 sequences for each of *trnH-psbA* and ITS. All sequences were submitted to the NCBI (**Table 1**).

**Table 3:** Sequence characteristics of four DNA markers and combinations of the markers.

	*rbcL*	*matK*	*trnH-psbA*	ITS
Universality of primers	Yes	Yes	Yes	Yes
Percentage PCR success (%)	96.15%	96.15%	100%	100%
Percentage sequencing success (%)	100%	100%	100%	100%
No. of species (no. of individuals)	19(50)	19(50)	19(52)	19(52)
No. of singleton species	9	9	9	9
Aligned length (bp)	1,207	835	379	589
Sequence length (bp)	1,207	835	299–379	587–589
No. of Parsimony-informative sites (%)	13(1.08)	17(2.04)	4(1.06)	50(8.49)
No. of variable sites (%)	24(1.99)	31(3.71)	16(4.22)	100(16.98)
No. of indels (length range)	0	0	11(1–25)	5(1–2)
Ranges of intraspecific distance	0	0–0.0014	0–0.0027	0–0.0116
Ranges of interspecific distance	0–0.0117	0–0.0207	0–0.0381	0–0.1161
Intraspecific distance (mean)	0	0.0004	0.0003	0.0032
Interspecific distance (mean)	0.0035	0.0064	0.0060	0.0323

No. of indels, the number of Insertions/Deletions; Interspecific distance (mean), the barcoding gap between species; Rate (%), percentage successful discrimination species calculated as the number of success discrimination species in relation to the total species; PWG, PWG-Distance method.

The aligned lengths of the *rbcL*, *matK*, *trnH-psbA*, and ITS barcode sequences in the dataset were 1,207, 835, 379, and 589 bp. Compared to the other markers, *rbcL* and *matK* were the most highly conserved, with lower percentages of variable sites and fewer indels. The lengths of the *rbcL* and *matK* sequences were always uniform. However, length variation existed for both the ITS (587–589 bp) and *trnH-psbA* (299–379 bp) markers. The number of variable sites was the highest for ITS markers (16.98%), followed by *trnH-psbA* (4.22%), *matK* (3.71%), and *rbcL* (1.99%). Eleven indels (1–25bp) were found at the *trnH-psbA* locus and five were found for ITS (1–2bp), while none were found for *matK* or *rbcL*.

### Interspecific and Intraspecific Variability

*rbcL* showed the lowest intraspecific and interspecific divergence as measured by the PWG-distance method, while ITS showed the highest interspecific divergence (0.1161), followed by *trnH-psbA* (0.0381) and *matK* (0.0207) (**Table 3**).

**Figure 2:**
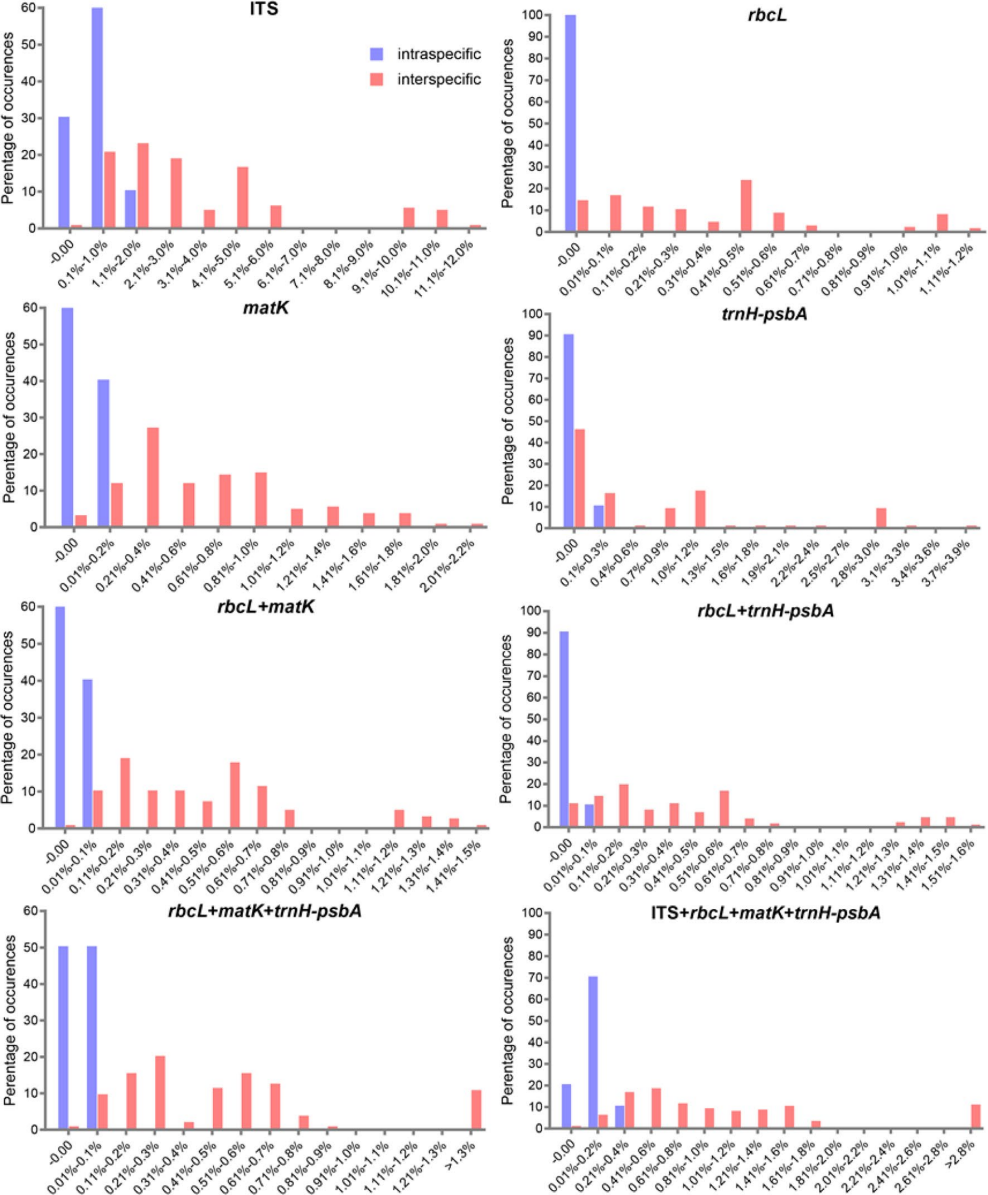
Histograms of the frequencies (y-axes) of pair wise intraspecific (blue bars) and interspecific (red bars) divergences based on the K2P distance (x-axes) for individual *rbcL matK*, *trnH*-*psbA*, and ITS markers and combined markers.

**Figure 3:**
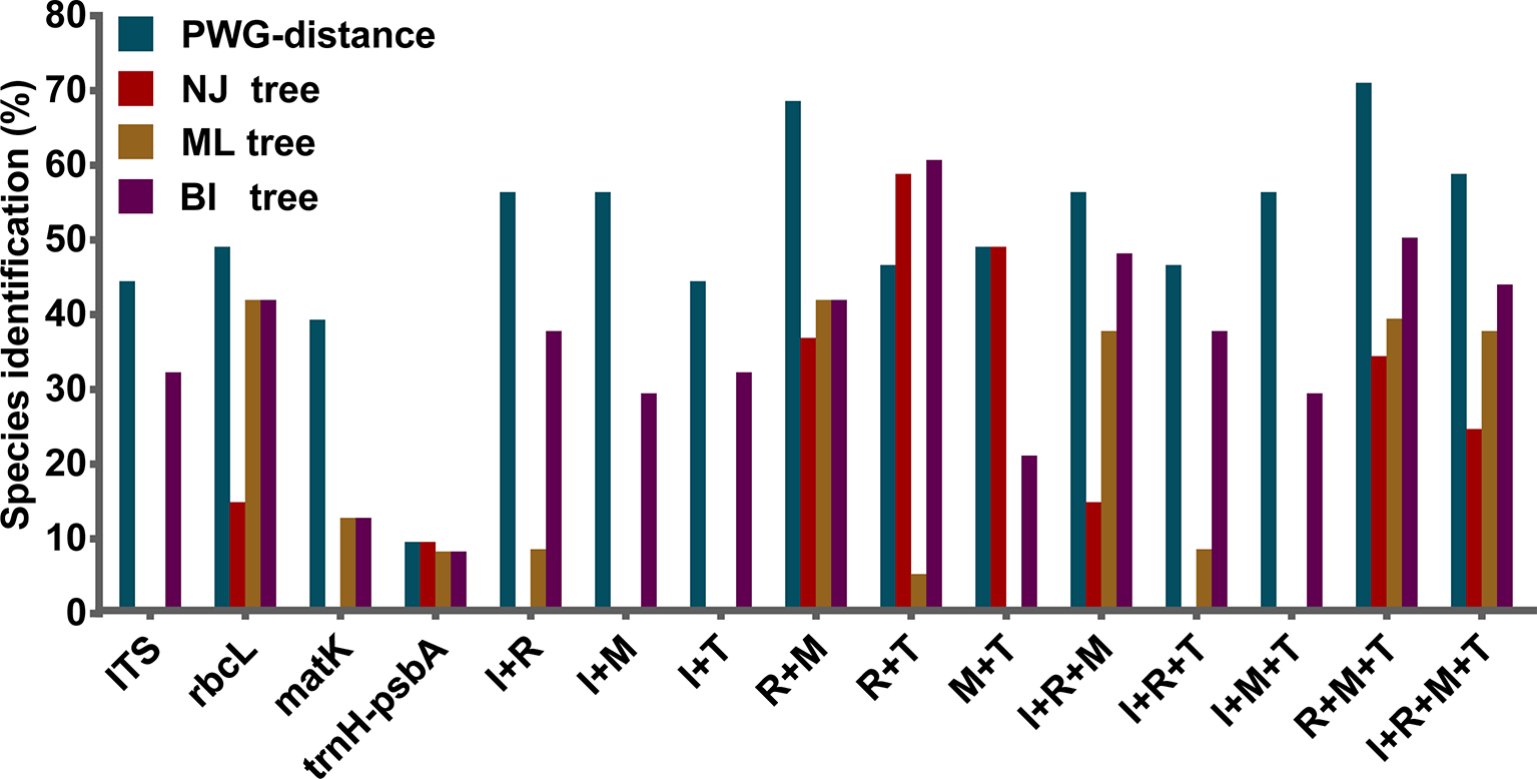
Species discrimination rates of all tested single- and multi-locus barcodes in *Pulsatilla, rbcL* (R), *matK* (M), *trnH*-*psbA* (T), and ITS (I).

We found overlaps for both single markers and combinations of the candidate loci, but we found no distinct barcoding gaps (**Figure 2**, **S1**). Among single barcodes, the *rbcL* marker showed the highest species resolution (48.78%), followed by ITS (44.19%), with *matK* and *trnH-psbA* showing the lower species resolution (14.63% and 9.30%, respectively). Of the eleven combinations, *rbcL*+*matK*+*trnH-psbA* (R+M+T) from the chloroplast genome exhibited the best species resolution (70.73%), followed by *rbcL*+*matK* (R+M) (68.29%), while *trnH-psbA*+ITS (T+I) had the lowest species resolution (44.19%) (**Figure 3**).

### Sequence Similarity (TAXONDNA)

TAXONDNA analyses using the “Best Match” and “Best Close Match” methods exhibited similar discrimination successes (**Table 4**). Among the four single barcodes, the ITS and *trnH-psbA* markers had the highest success rate (79.06%) for the correct identification of species, followed by *matK* (58.53%) and *rbcL* (48.78%), respectively. Among the eleven combinations, *matK*+ITS performed the best (83.72%). The *rbcL*+*matK*+ITS and *rbcL*+*matK*+*trnH-psbA*+ITS combinations had a success rate that was similar to *matK*+ITS.

**Table 4:** Ability of DNA barcode regions to discriminate species as assessed using TAXONDNA.

Region	Best match			Best close match		
	Correct	Ambiguous	Incorrect	Correct	Ambiguous	Incorrect
I	79.06%	16.27%	4.65%	79.06%	16.27%	4.65%
R	48.78%	51.21%	0.0%	48.78%	51.21%	0.0%
M	58.53%	39.02%	2.43%	58.53%	39.02%	2.43%
T	79.06%	16.27%	4.65%	79.06%	16.27%	4.65%
I + R	79.06%	16.27%	4.65%	79.06%	16.27%	4.65%
I + M	83.72%	9.3%	6.97%	83.72%	9.3%	6.97%
I + T	79.06%	13.95%	6.97%	79.06%	13.95%	6.97%
R + M	80.48%	17.07%	2.43%	80.48%	17.07%	2.43%
R + T	79.06%	16.27%	4.65%	79.06%	16.27%	4.65%
M + T	81.39%	9.3%	9.3%	81.39%	9.3%	9.3%
I + R + M	83.72%	9.3%	6.97%	83.72%	9.3%	6.97%
I + R + T	79.06%	13.95%	6.97%	79.06%	13.95%	6.97%
I + M + T	81.39%	9.3%	9.3%	81.39%	9.3%	9.3%
R + M + T	81.39%	9.3%	9.3%	81.39%	9.3%	9.3%
I + R + M + T	83.72%	9.3%	6.97%	83.72%	9.3%	6.97%

R, rbcL; M, matK; T, trnH-psbA; I, ITS.

### Species Discrimination (Tree-Building Methods)

The discrimination power for all markers and their combinations are shown in **Table 5**. Among the four single barcodes, the *rbcL* demonstrated the best discrimination power (NJ tree and BI tree: 48.78%), followed by ITS (BI tree: 39.02%), while *matK* and *trnH-psbA* both had lower discrimination power. When barcoding loci were combined, the *rbcL*+ITS combination had the highest resolution power (BI tree: 70.73%, NJ tree and ML tree: 58.54%), followed by *rbcL*+*matK*+*trnH-psbA*(BI tree: 58.54%) and *rbcL*+*matK*+ITS (BI tree: 56.10%). The core *rbcL*+*matK* combination recommended by COBL had relatively low resolution (NJ tree: 36.59%, ML tree and BI tree: 48.78%), and the *rbcL*+ITS combination was clearly better than all combinations (i.e. *rbcL*+*matK*+*trnH*-*psbA*+ITS; NJ tree: 24.39%, ML tree: 49.30%, BI tree:51.22%).

**Table 5:** Species discrimination rate of all tested single- and multi-locus barcodes in *Pulsatilla*.

Barcode	PWG (%)	NJ (%)	ML (%)	BI (%)
I	44.19	00.00	00.00	39.02
R	48.78	14.63	48.78	48.78
M	39.02	00.00	14.63	14.63
T	09.30	09.30	09.30	09.30
I + R	56.10	00.00	9.76	43.90
I + M	56.10	00.00	00.00	34.15
I + T	44.19	00.00	00.00	37.21
R + M	68.29	36.59	48.78	48.78
R + T	46.34	58.54	58.54	70.73
M + T	48.78	48.78	00.00	24.39
I + R + M	56.10	14.63	43.90	56.10
I + R + T	46.34	00.00	09.76	43.90
I + M + T	56.10	00.00	00.00	34.15
R + M + T	70.73	34.15	34.15	58.54
I + R + M + T	58.54	24.39	49.30	51.22

R, rbcL; M, matK; T, trnH-psbA; I, ITS. values in the parenthesis indicate species-level monophyly with bootstrap value ≥50. NJ, the Neighbor Joining (NJ) trees; BI, the Bayesian inference (BI) trees; ML, Maximum likelihood (ML) trees; PWG, the pair-wise genetic-based method.

**Figure 4:**
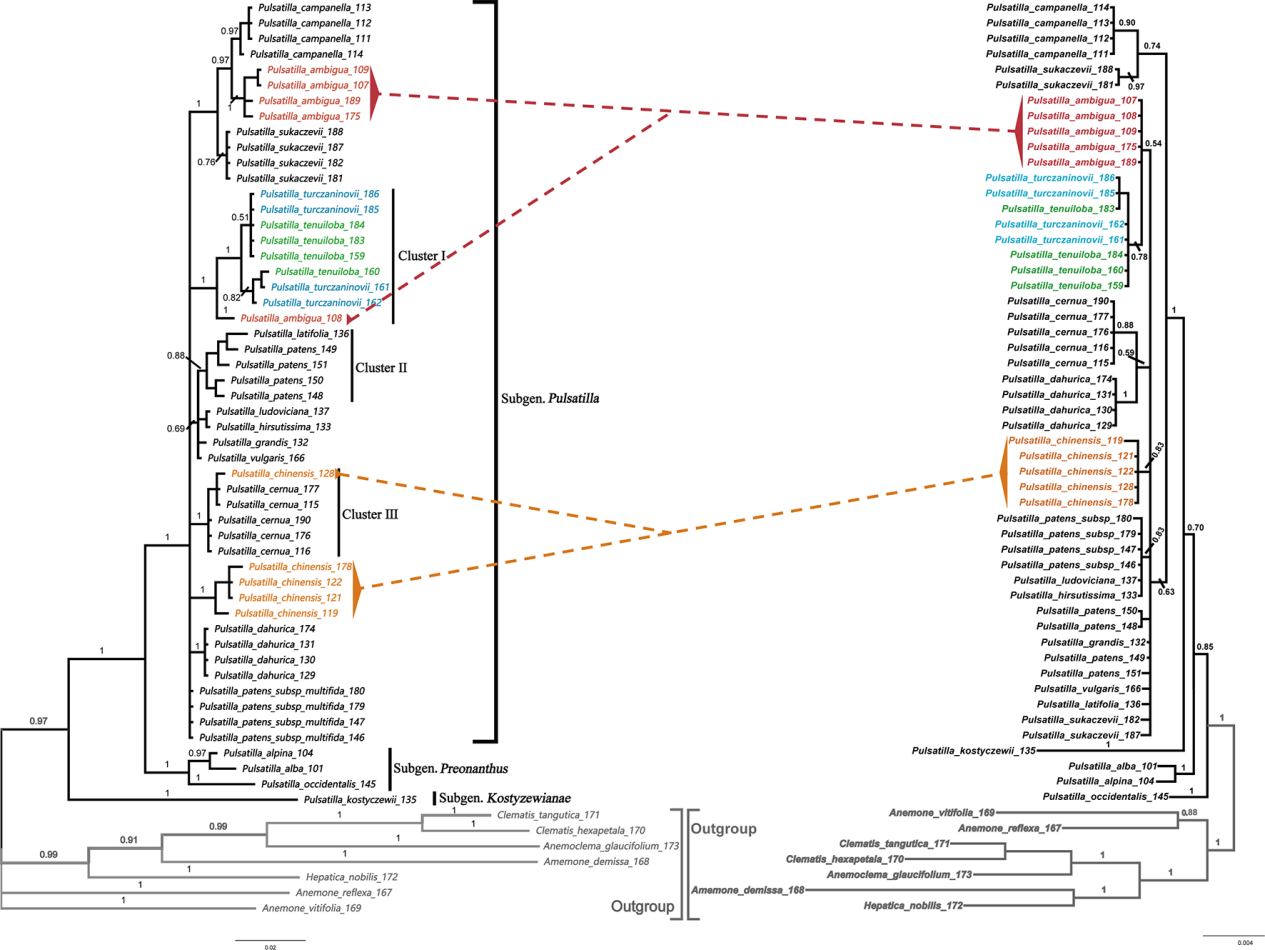
Bayesian inference (BI) trees based on ITS (left) and the combination of *rbcL*+*matK*+*trnH*-*psbA* sequences (right) in *Pulsatilla*. Bayesian posterior probabilities are given above branches.

The BI phylogenetic trees based on ITS (left) and chloroplast marker data (right) are shown in **Figure 4**, respectively. In all phylogenetic analyses, *Pulsatilla* formed a monophyletic clade with high bootstrap support (PP = 0.97/1.00), and all barcodes could discriminate between subgenera of *Pulsatilla*.

All samples in this study formed three subclades, corresponding to the three subgenera. In subgenus *Pulsatilla*, some species represented by two or more individuals formed monophyletic groups, e.g. *P. camoanella* and *P. dahurica*. However, several groups were mixed with individuals of other species, e.g. *P. turczaninovii* continually clustered with *P. tenuiloba*, and they are mixed with one individual from *P. ambigua* (cluster I); *P. latifolia*_136 was clustered with samples of *P. patens* (cluster II); *P. chinese*_128 was mixed with individuals of *P. cernua* (**Figure 4**, cluster III). We also found that *P. ludoviciana* and *P. hirsutissima* were mixed with individuals of *P. patens* subsp. *multifida*.

### Leaf Epidermis Micro-Morphology

**Figure 5:**
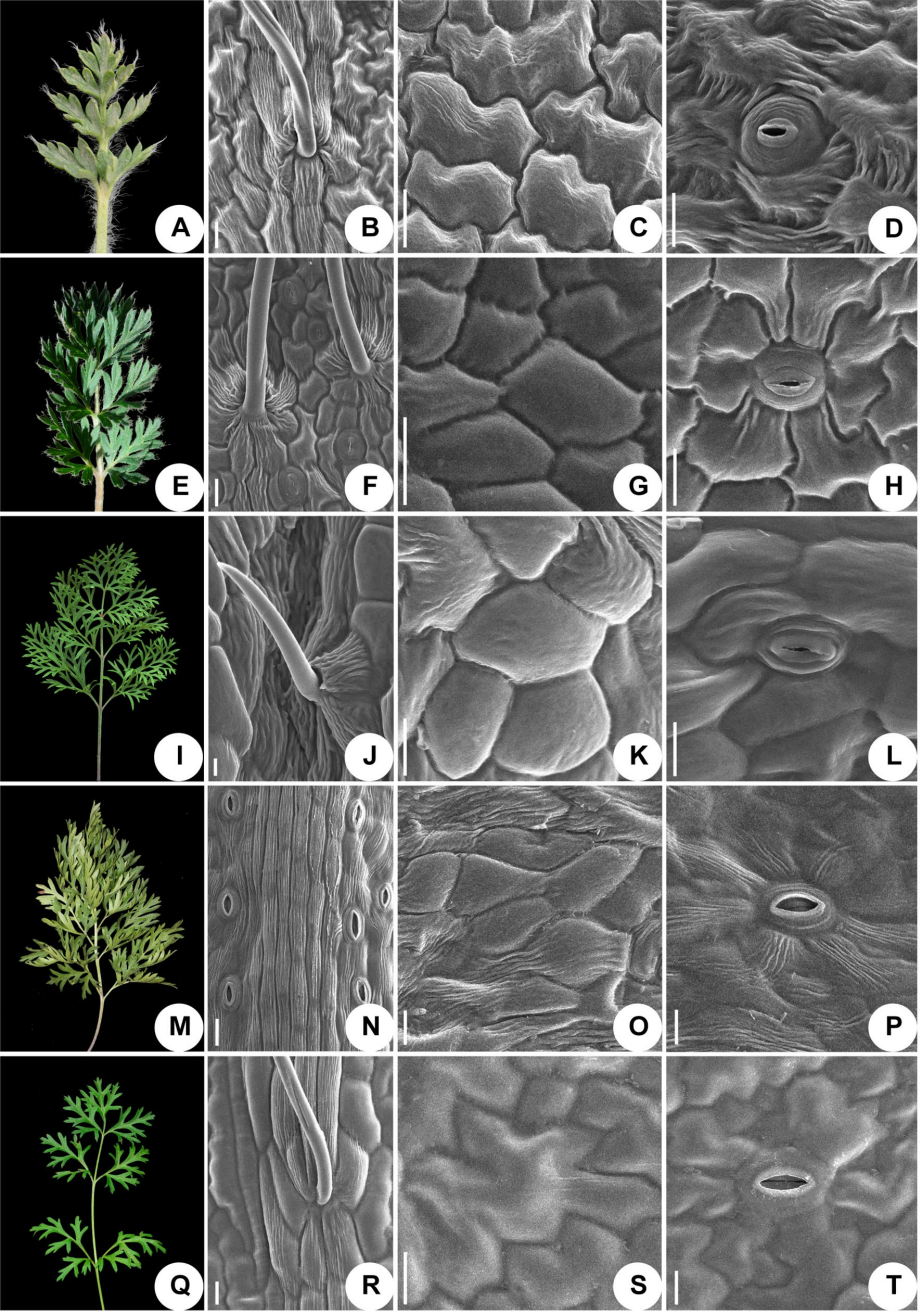
Micromorphological variation of leaf surfaces of *Pulsatilla* **(A–D)** *P. ambigua*; **(E–H)** *P. camoanella*; **(I–L)** *P. turczaninovii*; **(M–P)** *P. tenuiloba*; **(Q–T)** *P. vulgaris*. Scale bars: **B**–**D**, **F**–**H**, **J**–**L**, **N**–**P**, **R**–**T**, 200 µm.

**Figure 6:**
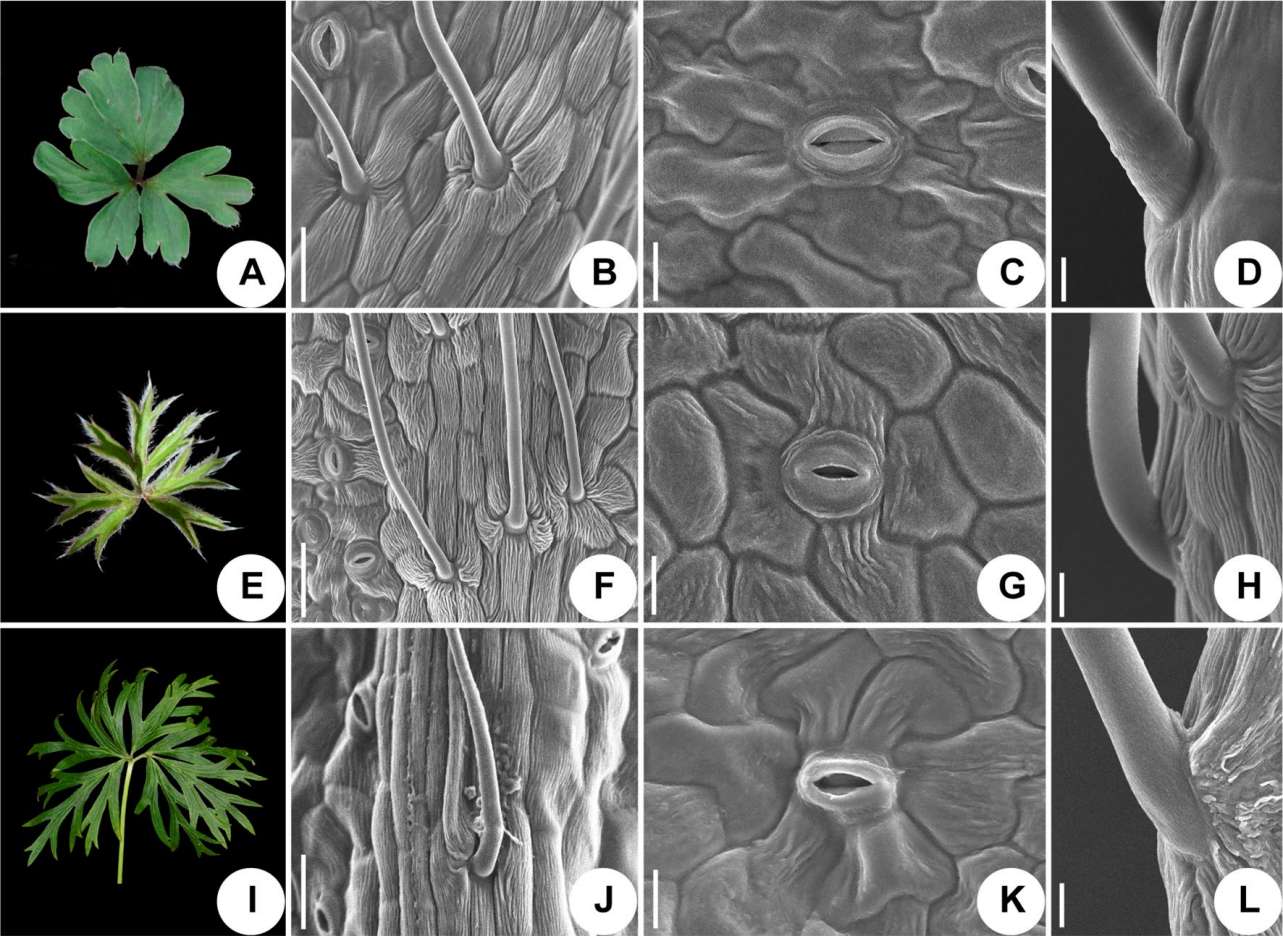
Micromorphological variation of leaf surfaces of *Pulsatilla*. **(A–D)** *P. chinensis*; **(E–H)** *P. patens*; **(I–L)** *P. patens* subsp. *multifida*; Scale bars: **B**, **F**, **J**, 0.5 mm; **C**, **G**, **K**, 200 µm; **D**, **H**, **L**, 100 µm.

We used scanning electronic microscopy to examine the leaf epidermis of eight species: *P. ambigua*, *P. camoanella*, *P. turczaninovii*, *P. tenuiloba*, *P. vulgaris* (**Figure 5**), *P. chinensis*, *P. patens*, and *P. patens* subsp. *multifida* (**Figure 6**). All observations were performed by imaging the back of the leaf. We found that the leaf epidermises of *P. ambigua* (**Figures 5A, B**), *P. camanella* (**Figures 5E, F**), *P. chinensis* (**Figures 6A, B**), and *P. patens* (**Figures 6E, F**) were pilose, and their trichomes were dense and long. The order of the density and length of trichomes were as follows: *P. ambigua* > *P. patens* > *P. chinensis* > *P. camanella*. In contrast, the leaf epidermises of *P. tenuiloba* (**Figures 5M, N**) was glabrous and those of *P. turczaninovii* (**Figures 5I, J**), *P. vulgaris* (**Figures 5Q, R**), and *P. patens* subsp. *multifida* (**Figures 6I, J**) showed sparsely short trichomes. The order of the density and length of trichomes were as follows: *P. patens* subsp. *multifida* > *P. turczaninovii* > *P. vulgaris* > *P. tenuiloba*.

The epidermal cells in all species were found to have a smooth polygonal shape on the epidermal walls, with the notable exceptions of *P. ambigua* (**Figure 5C**) and *P. vulgaris* (**Figure 5S**), which were found to have an irregular shape with sinuate anticlinal striation. Different species showed differences in stomatal organ type and cell shape. We found anomocytic (*P. ambigua*, **Figure 5D**; *P. vulgaris*, **Figure 5T**; *P. patens*, **Figure 6G**), actinocytic (*P. camanella*, **Figure 5H**; *P. tenuiloba*, **Figure 5P**; *P. chinensis*, **Figure 6C**; *P. patens* subsp. *multifida*, **Figure 6K**), and diacytic (*P. turczaninovii*, **Figure 5L**) stomata respectively.

## Discussion

### PCR and Sequencing Success

In this study, the short DNA sequences ITS and *trnH*-*psbA* had the best performance in PCR amplification and sequencing among the four barcode markers (quantified by amplification success). Moreover, successful sequencing rates for sequences ITS and *trnH-psbA* were over 90% for silica-dried samples but lower for herbarium specimens. These findings are consistent with many previous studies (Xu et al., 2015; Yan et al., 2015; Han et al., 2016). In addition, the varying lengths of insertions/deletions (indels) found at the *trnH*-*psbA* loci for different species provide important phylogenetic information and species discrimination power (Liu et al., 2011). Thus, sequence alignments of this region must be performed with great care to avoid overestimating substitution events.

The *rbcL* and *matK* genes are approximately 1,428 bp and 1,570 bp in length, respectively (Jing et al., 2011; Dong et al., 2012; Dong et al., 2014). The greatest problem with *rbcL* and *matK* was that it was difficult to amplify them from the degraded DNA isolated from old herbarium specimens, since the short lengths of remaining fragments hampered the extension phase of the PCR for these longer genes. Although some problems may be alleviated by using additional pairs of primers, the amplification and sequencing success rate of the old herbarium samples remained poor. Thus, we were not able to obtain all sequences for all herbarium samples.

### The Resolution of Chloroplast (*rbcL*, *matK*, and *trnH-psbA*) and Nuclear (ITS) Regions in *Pulsatilla*

An ideal DNA barcode should be universal, reliable, cost effective, and show considerable discriminatory power. Because none of the proposed single-locus barcodes perfectly meets all these criteria. It is generally necessary to use multi-locus barcodes for land plants (Kress and Erickson, 2007; Fazekas et al., 2008). Multi-locus barcodes can often improve the resolution rate of species identification (CBOL Plant Working Group, 2009;China Plant BOL Group, 2011).

In the present study, when evaluated alone, the species resolutions based on tree-building for the three chloroplast regions *rbcL*, *matK*, and *trnH-psbA* were 48.78, 14.63, and 9.30%, respectively. Low resolution phylogenetic trees made using the chloroplast regions mentioned above have been reported for other taxa, including *Curcuma* (2.3–7.9%) (Chen et al., 2015) and *Sisyrinchium* (5.11%–20.41%) (Alves et al., 2014). The inadequate resolution may be due to the lower substitution rates and lack of variation found in single plastid regions. Thus, we do not recommend single plastid regions as DNA barcodes for this the genus.

Among the candidate barcode genes, the Consortium for the Barcode of Life (CBOL) Plant Working Group suggested that *rbcL*, *matK*, and the *rbcL*+*matK* combination should be sufficient for a plant barcode, and that this combination should be supplemented with additional markers as required (Clegg, 1993; CBOL Plant Working Group, 2009; Hollingsworth et al., 2011; Dong et al., 2014). In addition, Kress et al. (2005) and Chase et al. (2007) proposed that *trnH-psbA* can be used in two-locus or three-locus barcode systems to improve resolution. For instance, two of the three combinations of the three chloroplast loci tested in this study, *rbcL*+*trnH-psbA* (BI tree: 70.73%, PWG: 46.34%) and *rbcL*+*matK*+*trnH-psbA* (PWG: 70.73%, BI tree: 58.54) exhibited higher discriminatory performance than any single marker. Consequently, this highlights the need to use chloroplast multi-locus barcodes (*rbcL*+*matK*+*trnH-psbA*) to improve the resolution of species identification in *Pulsatilla*.

The nuclear ITS region provided the highest inter-and intraspecific divergences (0.1161 and 0.0116, respectively) and had a higher success rate for the correct identification of species in TAXONDNA (Best match: 79.06%). However, as for the tree-building method, the discriminatory performance of ITS is not satisfactory, as its highest resolution is 39.02% (BI).

As evidenced by previous studies, the multi-locus barcode (*rbcL*+*matK*+*trnH-psbA*+ITS) is one of the combinations that demonstrated the highest species resolution rate, e.g., Aceraceae (90.5%) (Han et al., 2016), *Lysimachia* (95.5%) (Zhang et al., 2012), *Oberonia* (62.99%) (Li et al., 2016), *Rhodiola* (73.01%) (Zhang et al., 2015b) and Schisandraceae (75%) (Zhang et al., 2015a). However, in this study, addition of ITS to different kinds of combinations of chloroplast markers did not increase the resolution rate obviously (**Figure 4**). The resolution rate based on tree-building analyses was 51.22% for BI and 58.54% for PWG. In addition, we found no distinct barcoding gap. This phenomenon may be due to the one or more of several reasons. First, incomplete lineage sorting and non-homogeneous concerted evolution are likely to occur at the ITS locus (Liu et al., 2011; Liu et al., 2018; Wang et al., 2011; Wirta et al., 2016). Second, the three chloroplast regions (*rbcL*, *matK*, and *trnH-psbA*) cannot compensate for the drawbacks of ITS because they are sourced from a different genome. Although the nuclear genome (and the ITS region) is inherited biparentally, the chloroplast genome is inherited uniparentally. Thus, the chloroplast genome experiences more complete lineage sorting than the ITS locus does. Third, hybridizations may cause conflicts between ITS and chloroplast loci, as well as problematic results in ITS phylogeny due to the possibility of homogenization to paternal copies in some lineages and maternal copies in others.

A combination of DNA markers from different genomes—which have different modes of inheritance and conflicting phylogenies—can hinder our understanding of species delimitation and the evolutionary processes of speciation. Because of its myriad variable sites that can reliably distinguish species, resulting from a high mutation rate and rapid concerted evolution, we recommend ITS as a good single barcode for the genus *Pulsatilla*.

### Implications of DNA Barcoding and Micromorphological Characters for the Current Taxonomy of *Pulsatilla*

Phylogenetic identification and species recognition are foundationally important for biology (Moritz, 1994; Wirta et al., 2016). The results of the phylogenetic analyses performed in this study may shed some light on the identification and taxonomy of the genus *Pulsatilla* (**Figure 3**). Here, we found that *Pulsatilla* formed a monophyletic group with high support. Moreover, the three recognized subgenera — i.e. subg. *Pulsatilla*, subg. *Kostyczewianae*, and subg. *Preonanthus* (Grey-Wilson, 2014) — were resolved as distinct monophyletic groups, which is consistent with the recent phylogenetic result (Sramkó et al., 2019).

Within subgenus *Pulsatilla*, our analyses found that *P. camanella* and *P. ambigua* were resolved as sister to one another with high support. These two species share many common morphological characters, such as almost fully expanded leaves at anthesis, and dense, long trichomes. The flowers of both species nod before anthesis (Wang et al., 2001). However, during anthesis, the sepals of *P. camanella* can easily be distinguished from those of *P. ambigua* by color (blue-violet vs. dark violet). At the same time, the micro-morphological characters of the leaves are also different (smooth polygonal shape epidermal walls vs. irregular shape with sinuate anticlinal striation). Actinocytic and anomocytic stomata exist in both species, but most stomata in *P. camanella* are actinocytic, whereas most are anomocytic in *P. ambigua*. Thus, molecular data as well as micro-morphological characters can distinguish between these two species relatively well. Both types of evidence may be helpful to accurately identify specimens that are damaged or lack sufficient diagnostic characters.

In addition to its use in identifying specimens, DNA barcoding is also useful for resolving taxonomic uncertainty (Burns et al., 2008; Li et al., 2016). Our phylogenetic trees showed that *P. turczaninovii* always clustered with *P. tenuiloba*. They did not have distinct barcodes. The micro-morphological characters were also found to be the same, since both plants showed polygonal epidermal cells with striation, a dense distribution of stomata, and glabrous or sparsely short trichomes. In addition, the geographical distribution of these two species overlaps in Inner Mongolia. Taken together, these distinct lines of evidence collectively suggest that *P. turczaninovii* and *P. tenuiloba* are the same species.

The discovery of hybridization, introgression, and/or incomplete lineage sorting among species is another useful application of DNA barcoding (Wang et al., 1999, Xiao and Zhu, 2009). The chloroplast region is inherited maternally, but the nuclear genome, including the ITS region, is inherited biparentally (Alvarez and Wendel, 2003). Thus, if there are different results in different phylogenetic analyses from chloroplast and nuclear data, we speculate that these differences may be caused by hybridization and/or introgression among species, which could result in a non-monophyletic clade. In subg. *Pulsatilla*, we found several complex groups. The samples of *P. chinensis* and *P. cernua* in cluster III, were indistinguishable. In the Bayesian inference (BI) tree based on ITS sequences (**Figure 3**), the samples of *P. cernua* clustered in a clade along with sample *P. chinensis*128. However, in the Bayesian inference (BI) tree based on the combination of chloroplast sequences (**Figure 3**), sample *P. chinensis*128 clustered in a clade with all other samples of *P. chinensis*. *P. chinensis*is a widespread species and has a geographical range that covers that of *P. cernua*; in addition, sample *P. chinensis*128 was collected near populations of *P. cernua* in Jilin Province, China. Hybridization or introgression might have occurred during the speciation of *P. chinensis*is and *P. cernua*. A similar situation was also found for sample *P. ambigua*108 and cluster I (**Figure 3**), suggesting hybridization may have occurred between *P. ambigua* and *P. tenuiloba*/*P. turczaninovii*.

### Factors That Affect Species Discrimination

The resolution of the present study is relatively low compared to the 70% resolution reported by Fazekas et al. (2009) or other plant groups (Zhang et al., 2012; Zhang et al., 2015a; Zhang et al., 2015b; Han et al., 2016). Factors specific to the evolution of the *Pulsatilla* and/or the sampling strategy of this study may affect the ability to discriminate between species. Such factors include incomplete lineage sorting and hybridization, the rapid radiation of *Pulsatilla* species, the variation present at the barcode loci, and the sampling density used in this study.

Unlike animal species, many plant species have paraphyletic or polyphyletic origins due to the higher frequency of reticulate evolution, which is facilitated by hybridization and polyploidization (Rieseberg and Brouillet, 1994; Tate and Simpson 2003). Given that this is the case, barcoding based solely on plastid markers may not reliably distinguish species. For example, in our study, some species are resolved to paraphyletic groups, such as *P. patens*. In these cases, the use of nuclear DNA sequences (e.g. ITS markers) may improve the resolution among plant species because nuclear loci have higher overall synonymous substitution rates, thus making nuclear markers such as ITS more sensitive. In our study, *P. patens* samples 148, 149, 150, and 151 formed a monophyletic group with sample *P. latifolia* 136. However, these samples were not clustered together by chloroplast marker data (**Figure 3**).

## Conclusions

DNA barcoding promotes the development of high-resolution phylogenies (Guo et al., 2015). In this study, we selected nuclear ITS and three chloroplast barcodes to evaluate their suitability for use in classifying a comprehensive array of *Pulsatilla* samples. We found that ITS was the most efficient single-locus barcode, as marker data from this locus was able to identify accurately more than half of all *Pulsatilla* species. We also found that the combination of *rbcL*+*matK*+*trnH-psbA* was the most efficient multi-locus barcode. However, there is an upper limit to the information provided by the barcodes tested here and adding more fragments may not increase the discrimination power of the DNA barcoding process.

Due to hybridization and/or introgression into the genus *Pulsatilla*, supplementary use of other identification methods may assist DNA barcoding methods and permit more precise identification (Geißler et al., 2017; Valentini et al., 2017; Liu et al., 2018; Soares et al., 2018). In cases where *rbcL* and *matK* are difficult to amplify and/or perform unsatisfactorily, using the whole chloroplast genome as a marker represents a useful alternative to circumvent possible issues with gene deletion and low PCR efficiency (Huang et al., 2005). The idea of using whole chloroplast genomes, termed “super-barcoding”, to identify plant species, was proposed by Kane and Cronk (2008) and encouraged by Li et al. (2015). Recently, this approach has been used in practice in the feather grass genus *Hippophae* (Krawczyk et al., 2018). Future studies should aim to explore super-barcoding using next generation sequencing; such an approach may offer even more efficient discrimination of closely related species in the genus *Pulsatilla*. In addition, micro-morphological characters — e.g., pollen and seed grain, leaf and/or stem epidermis — may also provide useful supplementary data for plant identification. Combining molecular and micro-morphological data may also be an advisable strategy in the future.

## Data Availability Statement

All the sequences were deposited as GenBank: Accessions MK341798–MK342029.

## Author Contributions

LiaZ planned and designed the research. Q-JL, XW and J-RW performed experiments and analyzed data, Q-JL analyzed data, and Q-JL, XW, J-RW, NS, LinZ, Y-PM, Z-YC, LiaZ and DP wrote the manuscript.

## Funding

This project was supported by the Fundamental Research Funds for the Central Universities (No. 2452019068) and the National Nature Science Foundation of China (No. 31770200, 31300158, 31872710).

## Conflict of Interest

The authors declare that the research was conducted in the absence of any commercial or financial relationships that could be construed as a potential conflict of interest.

## Supplementary Material

Figure S1 Histograms of the frequencies (y-axes) of pair wise intraspecific (blue bars) and interspecific (red bars) divergences based on the K2P distance (x-axes) for combined markers of *rbcL matK*, *trnH-psbA*, and ITS.
