# Supplementary material for: Efficient Identification of Pulsatilla (Ranunculaceae) Using DNA Barcodes and Micro-Morphological Characters
Source: Front Plant Sci. 2019 Oct 9;10:1196. doi: 10.3389/fpls.2019.01196 (PMC6794950; doi:10.3389/fpls.2019.01196)
Supplement: Supplementary file 1 [file Table_1.doc]

**TABLE S1** List of segmented primers.

| **Region** | **Primer** | **Sequence (5'-3')** | **References** |
| --- | --- | --- | --- |
| *rbcL* | 1F | ATGTCACCACAAACAGAAAC | Fay et al.,1997 |
|  | 1R | GACATTCATAAACTGCTCT |  |
|  | 2F | ATTGAACAAGTATGGTCGTC |  |
|  | 2R | GGCAATAATGAGCCAAGCT |  |
|  | 3F | ATGCGAAGAAATGCTAAA |  |
|  | R | TCACAAGCAGCTAGTTCAGGACTC | Asmussen & Chase, 2001 |
| *matK* | 390F | CCTTATCATTTAGAGGAAGGAG | Cuénoud et al., 2002 |
|  | 1R | GTTGAGACCAAAAGTAAA |  |
|  | 2F | ACTATCCTATGGTTGTTC |  |
|  | 1326R | TCCTCCGCTTATTGATATGC | Cuénoud et al., 2002 |
