# Supplementary material for: Efficient Identification of Pulsatilla (Ranunculaceae) Using DNA Barcodes and Micro-Morphological Characters
Source: Front Plant Sci. 2019 Oct 9;10:1196. doi: 10.3389/fpls.2019.01196 (PMC6794950; doi:10.3389/fpls.2019.01196)
Supplement: Supplementary file 2 [file Table_2.doc]

**TABLE S2 The primer information and optimal PCR conditions used in this study.**

| Barcoding locus | Primer | Sequence (5’-3’) | PCR condition | References |
| --- | --- | --- | --- | --- |
| *rbcL* | 1F | ATGTCACCACAAACAGAAAC | 94◦C 3min, 30cycles (94◦C 1min, 48◦C 1min, 72◦C 1min), 72◦C 5min | Fay et al.,1997 |
|  | R | TCACAAGCAGCTAGTTCAGGACTC |  | Asmussen & Chase, 2001 |
| *matK* | 390F | CGATCTATTCATTCAATATTTC | 94◦C 3min, 26 cycles (94◦C 1min, 52◦C 1min, 72◦C 1min), 72◦C 7min | Cuénoud et al., 2002 |
|  | 1326R | TCTAGCACACGAAAGTCGAAGT |  | Cuénoud et al., 2002 |
| ITS | 5a F | CCTTATCATTTAGAGGAAGGAG | 94◦C 5min, 30cycles (94◦C 1min, 50◦C 1min, 72◦C 90s), 72◦C 10min | Stanford et al., 2000 |
|  | 4R | TCCTCCGCTTATTGATATGC |  | Stanford et al., 2000 |
| *trnH-psbA* | trnH2R | CGCGCATGGTGGATTCACAATCC | 94◦C 3min, 35 cycles (94◦C 30s, 54◦C 30s, 72◦C 40s), 72◦C 10min | Tate & Simpson, 2003 |
|  | psbAF | GTTATGCATGAACGTAATGCTC |  | Sang et al., 1997 |
