# Supplementary figures and images for: Efficient Identification of Pulsatilla (Ranunculaceae) Using DNA Barcodes and Micro-Morphological Characters
Source: Front Plant Sci. 2019 Oct 9;10:1196. doi: 10.3389/fpls.2019.01196 (PMC6794950; doi:10.3389/fpls.2019.01196)

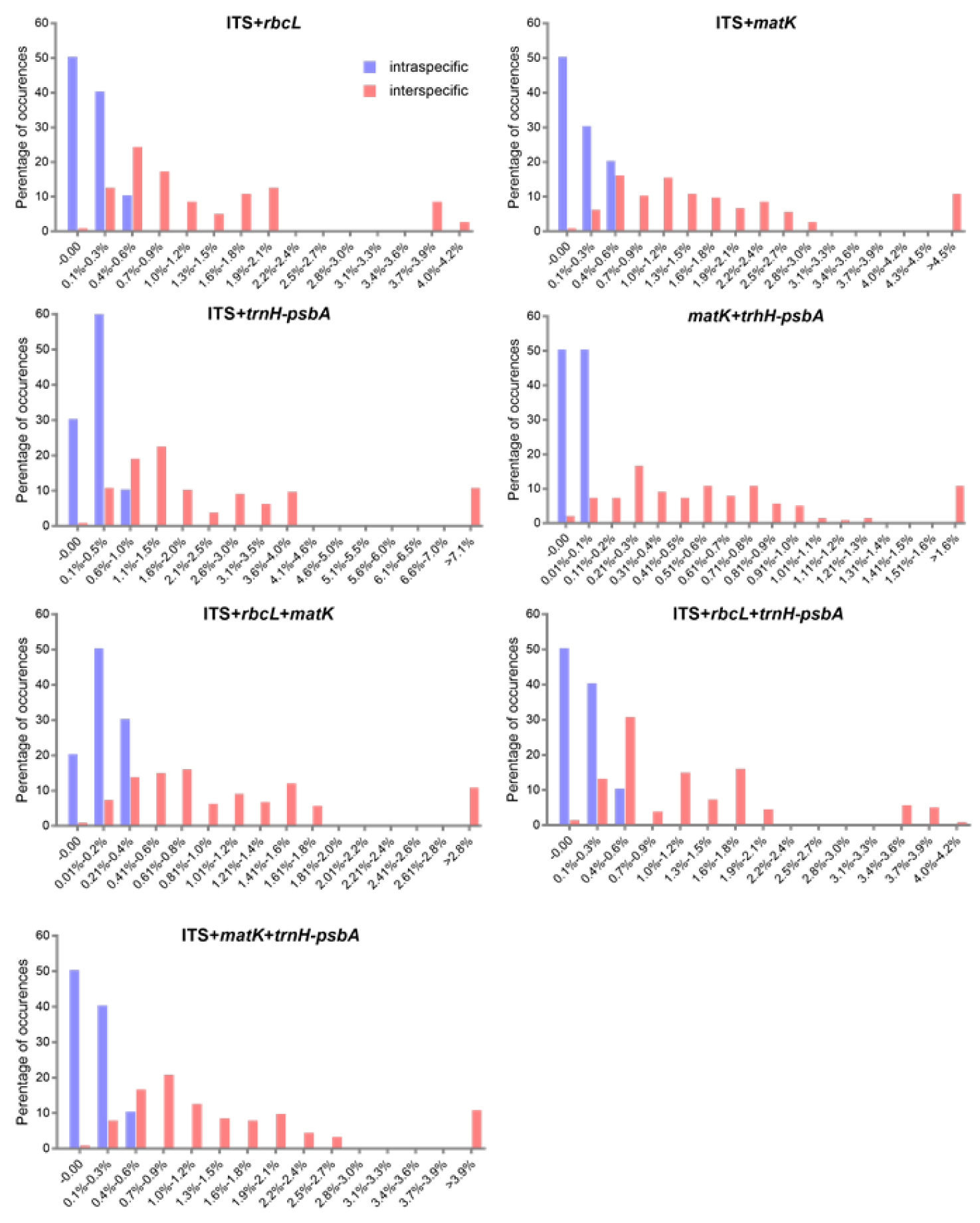

Supplement: Figure S1 — Histograms of the frequencies (y-axes) of pair wise intraspecific (blue bars) and interspecific (red bars) divergences based on the K2P distance (x-axes) for combined markers of rbcL matK, trnH-psbA, and ITS. [file Image_1.jpg]
